# Rational Design of Carbon Nitride Photoelectrodes with High Activity Toward Organic Oxidations

**DOI:** 10.1002/anie.202211587

**Published:** 2022-11-15

**Authors:** Carolina Pulignani, Camilo A. Mesa, Sam A. J. Hillman, Taylor Uekert, Sixto Giménez, James R. Durrant, Erwin Reisner

**Affiliations:** ^1^ Yusuf Hamied Department of Chemistry University of Cambridge Cambridge CB2 1EW UK; ^2^ Institute of Advanced Materials (INAM) Universitat Jaume I (UJI) 12006 Castelló de la Plana, Castellón Spain; ^3^ Department of Chemistry and Centre for Processable Electronics Imperial College London London W12 0BZ UK

**Keywords:** Carbon Nitride, Organic Oxidation, Photoanodes, Photoelectrochemistry, Spectroscopy

## Abstract

Carbon nitride (CN_*x*_) is a light‐absorber with excellent performance in photocatalytic suspension systems, but the activity of CN_*x*_ photoelectrodes has remained low. Here, cyanamide‐functionalized CN_*x*_ (^NCN^CN_*x*_) was co‐deposited with ITO nanoparticles on a 1.8 Å thick alumina‐coated FTO electrode. Transient absorption spectroscopy and impedance measurements support that ITO acts as a conductive binder and improves electron extraction from the ^NCN^CN_*x*_, whilst the alumina underlayer reduces recombination losses between the ITO and the FTO glass. The Al_2_O_3_|ITO : ^NCN^CN_*x*_ film displays a benchmark performance for CN_*x*_‐based photoanodes with an onset of −0.4 V vs a reversible hydrogen electrode (RHE), and 1.4±0.2 mA cm^−2^ at 1.23 V vs RHE during AM1.5G irradiation for the selective oxidation of 4‐methylbenzyl alcohol. This assembly strategy will improve the exploration of CN_*x*_ in fundamental and applied photoelectrochemical (PEC) studies.

## Graphical abstract

Carbon‐nitride‐based photoelectrodes were produced by co‐deposition of a blend of cyanamide‐functionalized carbon nitride (^NCN^CN_x_) and indium tin oxide (ITO) nanoparticles on top of thin, alumina‐coated conducting FTO‐glass. The composite photoanodes display benchmark photocurrents with a range of different alcohol substrates, a low onset potential, and an incident photon‐to‐electron conversion efficiency (IPCE) up to 60 % at 350 nm.

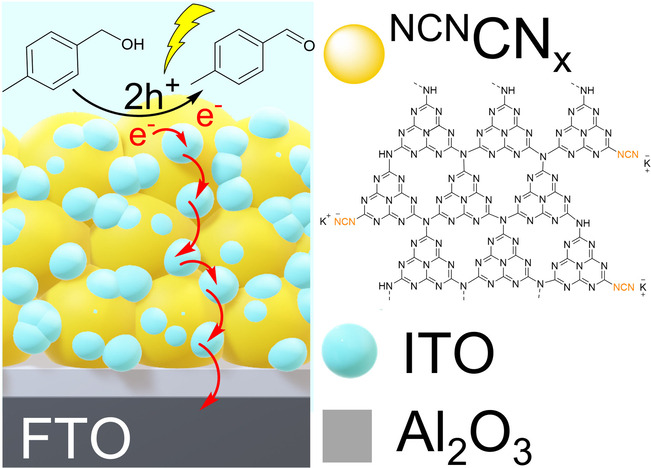

## Introduction

Carbon nitride (CN_*x*_) has emerged in recent years as a promising heterogeneous photocatalyst with the prospect of replacing homogeneous precious metal catalysts.[^1^, ^2^] CN_*x*_ materials offer high stability in many common solvents as well as facile and scalable fabrication by thermal polymerization of abundant and inexpensive nitrogen‐rich precursors, such as melamine, dicyandiamide, cyanamide, urea, and thiourea.[^3^, ^4^] This environmentally benign semiconductor has a band gap of 2.7 eV and band positions that can be easily tuned by chemical or structural modification, allowing it to photocatalyze reactions such as water oxidation, proton and CO_2_ reduction, and hydrogen peroxide production.[^5^, ^6^, ^7^] A quickly growing collection of organic transformations is also being reported, with examples being the selective oxidation of alcohols to aldehydes or ketones, C−C or C−heteroatom bond formation, cyclization, and arene functionalization.[^3^, ^8^, ^9^, ^10^, ^11^, ^12^]

The use of CN_*x*_ in PEC systems is being explored for several reasons: (i) PEC systems allow for practical device assembly and easy pairing with a second light absorber in tandem PEC cells.^[13]^ (ii) The use of electrodes in separate compartments allows for easier separation of products when compared to dispersed photocatalysts.^[14]^ (iii) Physical separation of two half reactions in two compartments limits back‐reactions, a major efficiency bottleneck in solution and suspension systems.^[15]^ (iv) Photoelectrodes can be used as a simplified platform to investigate reaction mechanisms and substrate breakdown processes for fundamental mechanistic studies of the half‐reaction of interest. The externally applied potential can be employed to manipulate the reaction mechanism and kinetics for tuning product selectivity, which is unperturbed by the redox chemistry occurring at the counter electrode.^[16]^

However, some of the intrinsic characteristics of CN_*x*_ have challenged the transition from dispersed photocatalysts to PEC systems.^[17]^ The low conductivity and high recombination rates restrict PEC performance of CN_*x*_ films.[^18^, ^19^] Immobilization on an electrode often results in unstable and non‐uniform photoelectrodes due to the large particle size, low surface area, and weak adhesion of CN_*x*_ to the electrode surface,^[20]^ despite various deposition techniques (e.g., thermal and chemical vapor condensation,[^20^, ^21^, ^22^, ^23^, ^24^, ^25^, ^26^] direct growth,^[28]^ seed‐growth,^[29]^ doctor‐blading,[^30^, ^31^] and sol‐gel processing^[32]^) being reported. Although CN_*x*_ can reach higher photocurrents when paired with another photoabsorber,[^33^, ^34^, ^35^] the as‐synthesized CN_*x*_ photoanodes on their own only exhibit photocurrent densities up to ≈660 μA cm^−2^ with a sacrificial substrate (i.e., triethanolamine) at an applied potential of 1.23 V vs RHE during AM1.5G irradiation (Figure S1). Such currents are low compared to other well‐established metal oxide photoanodes such as BiVO_4_, WO_3_, and α‐Fe_2_O_3_, which can reach stable and reproducible photocurrents of 3 mA cm^−2^ at 1.23 V vs RHE during AM1.5G irradiation even for water oxidation (without an external hole scavenger).[^2^, ^36^] Multiple combined strategies to overcome these limitations for CN_*x*_ electrodes are under investigation, with metal‐doping, monomer modification and cocatalyst immobilization being the preferred ones.^[37]^ However, there remains overall a lack of basic understanding of the intrinsic processes occurring within a CN_*x*_ electrode, which prevents the rational design of photoelectrodes with high current densities.

**Figure 1:**
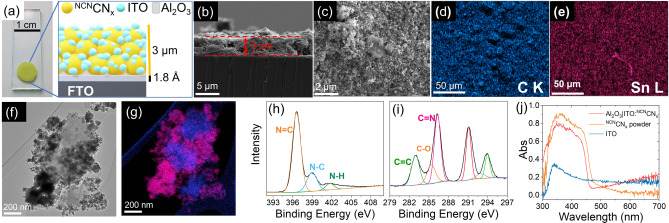
a) Picture of Al_2_O_3_|ITO : ^NCN^CN_x_ electrode, area 0.25 cm^2^, with a schematic representation of the different components. b) Cross‐section and c) top‐view SEM images of an Al_2_O_3_|ITO : ^NCN^CN_*x*_ photoanode. Top‐view SEM EDS mapping of Al_2_O_3_|ITO : ^NCN^CN_*x*_ electrodes showing the elemental distribution of d) carbon and e) tin. Samples were sputter‐coated with a 10 nm layer of Cr prior to measurement. f) TEM image of ITO : ^NCN^CN_*x*_, scratched from an electrode, and g) TEM EDS mapping showing carbon (blue) and tin (pink) elemental distribution. XPS spectra of h) N 1s and i) C 1s edges for the Al_2_O_3_|ITO : ^NCN^CN_*x*_ electrodes. j) UV/Vis spectra of Al_2_O_3_|ITO : ^NCN^CN_*x*_ electrodes (red trace), ^NCN^CN_*x*_ powder (orange trace), and ITO nanoparticles (blue trace).

In this work, a cyanamide‐functionalized CN_*x*_ (^NCN^CN_*x*_) photoelectrode was rationally designed to improve device conductivity and charge‐transfer efficiencies to eliminate performance bottlenecks (Figure 1). A poly(heptazine imide) (PHI) ionic carbon nitride, ^NCN^CN_*x*_,^[38]^ was selected because of its established charge‐separation efficiency, high activity, and selectivity in suspension for alcohol oxidation.[^19^, ^39^] The co‐deposition of this poly‐heptazine polymer with indium tin oxide (ITO) nanoparticles on an atomic‐layer‐deposited (ALD) alumina‐coated FTO glass electrode led to a photoanode exhibiting a benchmark photocurrent density when selectively oxidizing 4‐methylbenzyl alcohol (4‐MBA), a model reagent for organic oxidations (Figure 1a, Figure S1). This versatile photoanode exhibits high activity toward several oxidation reactions, including oxidation of ethylene glycol, glycerol, and simple alcohols such as methanol and ethanol. Spectroscopic and PEC studies reveal that the benchmark 4‐MBA oxidation current densities are due to improved electron extraction from the photoexcited ^NCN^CN_*x*_ to the FTO contact.

## Results and Discussion

The ^NCN^CN_*x*_ material was synthesized from melamine at 550 °C, followed by post‐synthetic modification with potassium thiocyanate (weight ratio 1 : 2), according to literature procedures.[^39^, ^40^] Attenuated total reflection infrared (ATR‐IR) spectroscopy confirmed the presence of the cyanamide functionality, identified by a characteristic vibration at 2177 cm^−1^ (C=N stretch, Figure S2a). Diffuse reflectance UV/Vis spectroscopy confirmed the enhanced visible‐light absorption (λ<460 nm, Figure S2b) caused by the post‐functionalization. For the preparation of the composite electrode, a total mixture of 5 wt % of ^NCN^CN_*x*_ and ITO nanoparticles (diameter<50 nm, surface area 47 m^2^ g^−1^) was sonicated in ethanol. The ^NCN^CN_*x*_ : ITO weight ratio was optimized, with a one‐to‐one wt % ratio giving the highest photocurrent response (Figure S3a). Two layers of 5 μL each were drop‐casted into a 0.25 cm^2^ circular mask attached to alumina‐coated FTO‐glass. Three different thicknesses of Al_2_O_3_ were studied (1.8, 2.7, and 3.6 Å), with the thinnest one chosen for further investigation as it produced the highest photocurrent increase (Figure S3b). The samples were further annealed at 250 °C for one hour under inert atmosphere (Ar) to ensure solvent evaporation and binding of the two components. Titanium oxide (TiO_2_) nanoparticles were also studied as an electron collection scaffold, but the overall photoelectrode activity was lower compared to ITO (Figure S4a).

Transmission electron microscopy (TEM) of the Al_2_O_3_|ITO : ^NCN^CN_*x*_ photoelectrodes shows the homogeneous blending of ITO nanoparticles within the carbon nitride matrix (Figure 1, Figure S5), leading to improved adhesion of ^NCN^CN_x_ to the conductive support (Figure S6). Scanning electron microscopy (SEM) confirms that ^NCN^CN_*x*_ agglomerates are connected by a network of smaller ITO particles, giving a relatively uniform film thickness of 3.0±0.3 μm (Figure 1). Top‐down energy‐dispersive X‐ray spectroscopy (EDS) mapping further validates the uniform distribution of C, N, In, and Sn throughout the film (Figure 1). No Al was detectable on the surface of the films, suggesting that the bottom Al_2_O_3_ layer does not mix with the catalytic mixture during the annealing process.

X‐ray photoelectron spectroscopy (XPS) confirmed that the surface properties of ^NCN^CN_*x*_ were largely unaffected by the co‐deposition with ITO nanoparticles and the annealing step (Figure 1). The high‐resolution N 1s and C 1s spectra of the Al_2_O_3_|ITO : ^NCN^CN_*x*_ photoanodes were nearly identical to the ^NCN^CN_*x*_ powder and slightly shifted from literature observations.[^39^, ^41^] The *sp* ^2^ carbon (286.3 eV) and nitrogen (396.5 eV) signals confirmed the heptazine core of the material. XPS spectra of Sn, In 3d, and O 1s levels allowed confirmation of the unmodified chemical nature of ITO nanoparticles (Figure S7).^[42]^ Furthermore, the diffuse reflectance UV/Vis spectrum of the electrode matches that of the ^NCN^CN_*x*_ powder, confirming the attachment of the material to the conductive support with no change in absorption related to the synthetic procedure (Figure 1). The increased baseline at λ>460 nm can be attributed to scattering by the ITO nanoparticles. The absorption onset in both cases at about 450 nm corresponds to a band gap of 2.7 eV (Figure S8).

**Figure 2:**
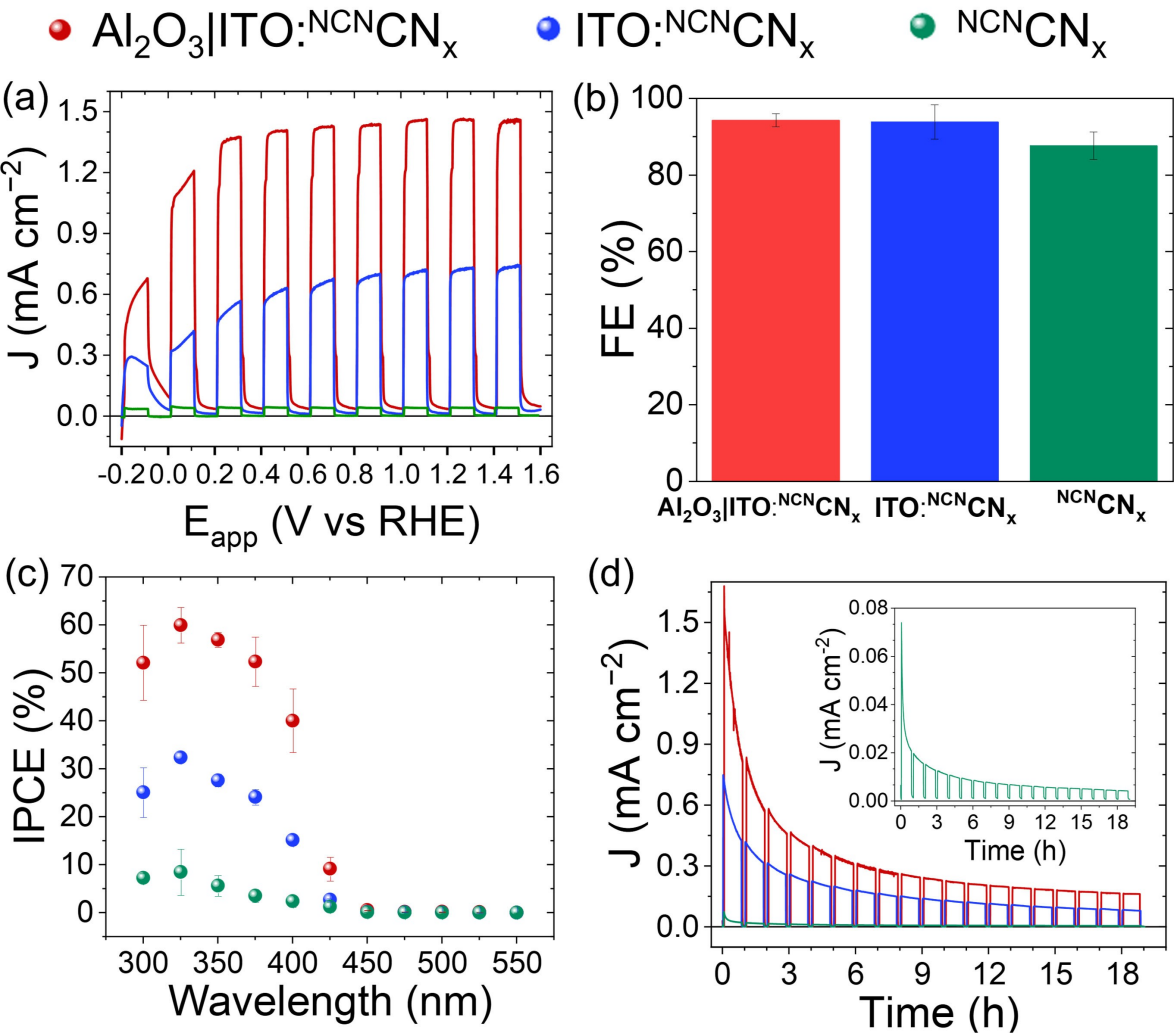
a) Linear sweep voltammetry scans, b) Faradaic efficiencies (FE), c) IPCE measurements, and d) chronoamperometry scans of Al_2_O_3_|ITO : ^NCN^CN_*x*_ (red trace), ^NCN^CN_*x*_‐ITO (blue trace), and ^NCN^CN_*x*_ (green trace, inset) photoelectrodes (area 0.25 cm^−2^). PEC, FE, and IPCE conditions: 50 mM 4‐MBA in 0.1 M Na_2_SO_4_ (pH 7, 9 mL) at 1.23 V vs RHE, scan rate (10 mV s^−1^), chopped (10 sec on/off for LSVs and 50 min on/10 min off for chronoamperometry) simulated solar light (AM 1.5G, 100 mW cm^−2^), N_2_ atm, 25 °C, front illumination.

The PEC properties of the Al_2_O_3_|ITO : ^NCN^CN_*x*_ electrodes were studied under chopped simulated solar light (AM 1.5G, 100 mW cm^−2^) at pH 7 in aqueous Na_2_SO_4_ (0.1 M) electrolyte solution at 25 °C. All the experiments were performed in a single‐compartment cell in three‐electrode configuration, with Ag/AgCl as the reference and a platinum wire as the counter electrode. 4‐MBA was selected as the organic substrate for PEC oxidation because it was previously shown to produce *p*‐tolualdehyde (4‐methylbenzaldehyde) with high selectivity in aqueous ^NCN^CN_*x*_ suspensions.[^43^, ^44^, ^45^] All measurements were performed by illuminating the sample from the front since back illumination resulted in a current drop of 23±1 % (Figure S9a). Al_2_O_3_|ITO : ^NCN^CN_*x*_ showed a photocurrent response even at negatively applied potentials, with an estimated onset potential of approximately −0.4 V vs RHE (Figure S10), which matches state‐of‐the‐art materials such as α‐Fe_2_O_3_ for alcohol oxidation.^[46]^ In contrast to other carbon‐nitride‐based photoanodes with a similar low photocurrent onset,[^32^, ^47^] no positive photodoping effect was observed after pre‐illumination (Figure S9b) and light soaking experiments resulted in a photocurrent drop of 20±0.5 %. Furthermore, the Al_2_O_3_|ITO : ^NCN^CN_*x*_ photoelectrodes displayed a record current density for alcohol oxidation, reaching 1.4±0.2 mA cm^−2^ (Figure 2a) at 1.23 V vs RHE in a 4‐MBA solution (50 mM), with a Faradaic efficiency (FE) higher than 95 % (Figure 2b), as quantified by HPLC. This corresponds to 22.3±2.5 % conversion of 4‐MBA to the aldehyde product after 18 h chronoamperometry. No substantial change of pH was recorded after linear sweep voltammetry (LSVs) or chronoamperometry (pH 7.1±0.1). The incident photon‐to‐current efficiency (IPCE) at 1.23 V vs RHE reached 60.0±3.6 % using *λ*=325 nm irradiation (Figure 2c).

The removal of the alumina underlayer resulted in a halving of the photocurrent response and the IPCE maximum value at *λ*=325 nm, reaching 0.7 mA cm^−2^ and 32 %, respectively (Figure 2a,c blue data). By further removing the ITO nanoparticles from the drop casting blend, the photocurrent and IPCE dropped by a further ≈7‐fold (Figure 2a, c, green data). Although the ITO : ^NCN^CN_*x*_ and ^NCN^CN_*x*_ photoanodes displayed a reduced photocurrent, they retained a FE higher than 87 % (Figure 2b), corresponding to 12±8 % and 0.5±0.1 % conversion, respectively. The Al_2_O_3_|ITO : ^NCN^CN_*x*_ electrode could not act as an electrocatalyst and the conversion of 4‐MBA was less than 0.4 % in the dark, measured after 18 h chronoamperometry at 1.23 V vs RHE (Table S1). A control experiment with an ITO‐only electrode (no ^NCN^CN_*x*_) immersed in the electrolyte solution showed no activity (Table S1, Figure S4b), confirming the innocence of the ITO nanoparticles towards the studied organic oxidation. Wiring the Al_2_O_3_|ITO : ^NCN^CN_*x*_ photoanode to a Pt cathode (2‐electrode set‐up) enabled operation without an applied external bias and allowed for 4.6±0.5 % conversion (Table S1).

Durability studies by chronoamperometry revealed an exponential decrease of the photocurrents over time (Figure 2d) for all three photoelectrodes. Under illumination at an applied potential of 1.23 V vs RHE, the Al_2_O_3_|ITO : ^NCN^CN_*x*_ photoanode lost 70±3 % of the initial photocurrent after 4 h, and 90±2 % after 16 h (Figure 2d). This substantial current drop may be related to various phenomena: leaching of the ^NCN^CN_*x*_ from the FTO glass into the bulk solution, partial photooxidation of the ^NCN^CN_*x*_ surface, or surface deactivation caused by (photo)deposition of side‐products (i.e., aldehyde conjugated oligomers). SEM images of the post‐mortem film (Figure S11) show an increased number of cracks on the electrode surface, supporting ^NCN^CN_*x*_ detachment from the conductive glass support. Furthermore, the UV/Vis spectrum of the ^NCN^CN_*x*_ film shows a decrease in absorbance after 18 h illumination (Figure S12a). The same substantial current drop was observed after chronoamperometry was performed without external potential, or in the dark with an applied potential (Figure S12b). In addition, the initial photocurrent could not be recovered after washing the electrodes with organic solvent (i.e., ethanol or acetonitrile) to remove possible side‐products deposited on the surface, and then placing them in a new 4‐MBA solution (50 mM). Thus, ^NCN^CN_*x*_ leaching may be the most plausible explanation for the photocurrent drop over time.

**Figure 3:**
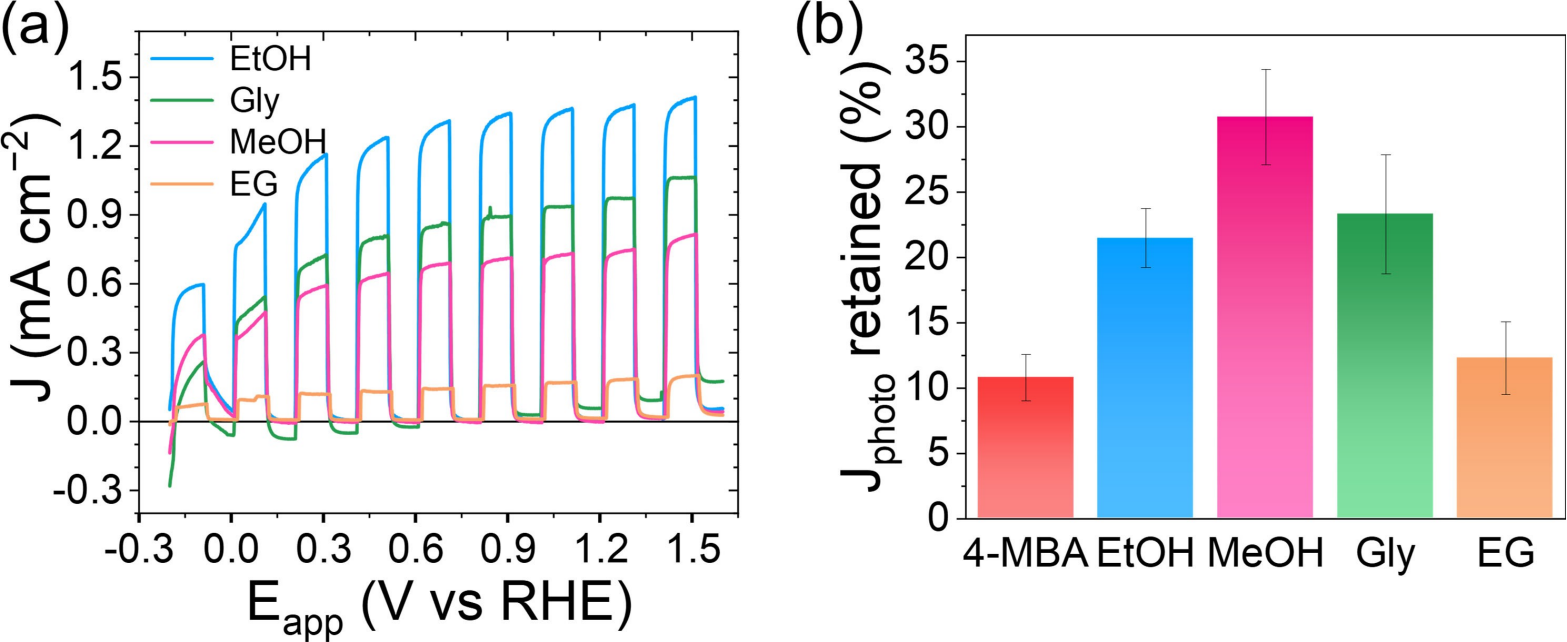
a) Linear sweep voltammetry scans of Al_2_O_3_|ITO : ^NCN^CN_*x*_ photoelectrodes (area 0.25 cm^−2^) oxidizing methanol (pink trace), ethanol (blue trace), glycerol (green trace), and ethylene glycol (orange trace). b) Percentage of photocurrent retained after 18 h of chopped illumination (50 min on/10 min off), at 1.23 V vs RHE. Conditions: 10 % v/v of alcohol in 0.1 M Na_2_SO_4_ (9 mL), and 50 mM solution of 4‐MBA or EG, simulated solar light (AM 1.5G, 100 mW cm^−2^), N_2_ atm, 25 °C, front illumination.

We also studied the electrodes’ versatility towards the oxidation of various alcohols such as glycerol (the main by‐product of biodiesel production),^[48]^ ethylene glycol (EG, the co‐monomer in polyethylene terephthalate (PET) and a model molecule for plastic photoreforming),^[49]^ and methanol and ethanol (small simple alcohols accessible from plant biomass). As shown in Figure 3a, the photocurrent response of the optimized photoanodes remained between 0.7–1.4 mA cm^−2^ at 1.23 V vs RHE for glycerol, ethanol, and methanol; whereas in the case of EG the photocurrent response reached 0.25 mA cm^−2^ at 1.23 V vs RHE. In general, the oxidation reactions were less selective with these substrates, and mixtures of different products were detected (Figures S13 and S14), in accordance with previous literature studies.[^41^, ^50^] The Al_2_O_3_|ITO : ^NCN^CN_x_ photoanode performance was further confirmed with triethanolamine (TEOA), a widely employed sacrificial electron donor. The photoelectrode exhibited a photocurrent of 0.95 mA cm^−2^ when immersed in a 10 % v/v aqueous TEOA solution (Figure S15). Interestingly, when oxidizing the various 10 % v/v aqueous alcohol solutions, between 22–30 % of the starting photocurrents could be retained after 18 h of light irradiation at an applied potential of 1.23 V vs RHE (Figure 3b), more than double than for 4‐MBA oxidation.

**Figure 4:**
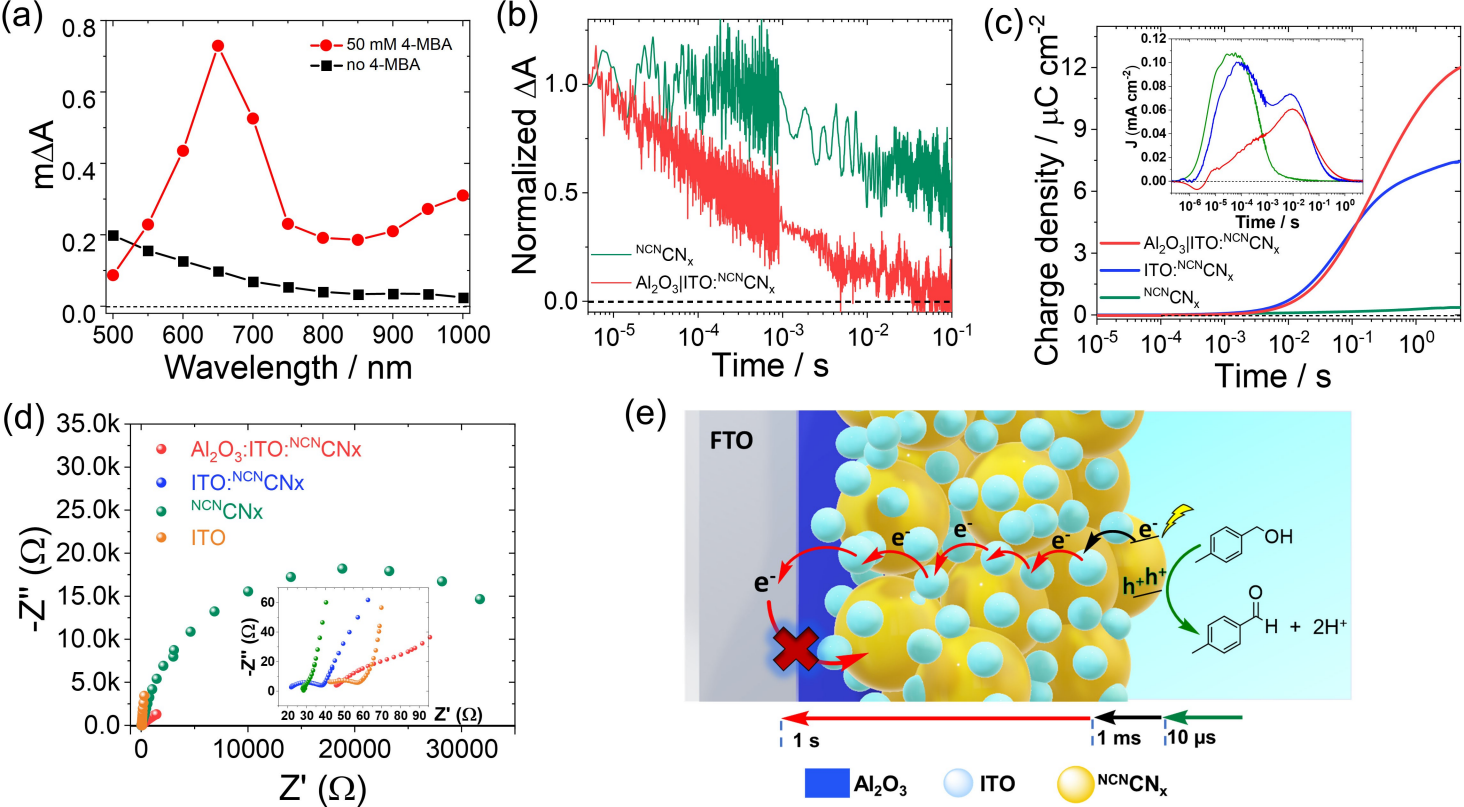
a) Photoinduced absorption (PIA) spectra of an Al_2_O_3_|ITO : ^NCN^CN_*x*_ electrode in 0.1 M Na_2_SO_4_ solution, with and without 4‐MBA (50 mM). b) Transient absorption kinetics, probed at 650 nm, for ^NCN^CN_*x*_ and Al_2_O_3_|ITO : ^NCN^CN_*x*_ samples after front excitation with a 355 nm laser pulse (9 ns width, 540 μJ cm^−2^). c) Total extracted charge density as a function of time after excitation, calculated by integrating the transient photocurrents (inset) after front excitation with a 355 nm laser pulse (9 ns width, 540 μJ cm^−2^). Samples are held at 1.2 V vs RHE in 50 mM 4‐MBA (0.1 M Na_2_SO_4_). d) Nyquist plot measured by PEIS of the Al_2_O_3_|ITO : ^NCN^CN_*x*_ electrode and the respective ITO : ^NCN^CN_*x*_, ^NCN^CN_*x*_ and ITO blanks at 0 V vs RHE. Conditions: 50 mM 4‐MBA solution under simulated solar light (AM 1.5G, 100 mW cm^−2^) N_2_ atm, 25 °C, front illumination. (e) Representation of the timescale of electron‐transfer processes in Al_2_O_3_|ITO : ^NCN^CN_*x*_.

We subsequently performed mechanistic and kinetic studies to elucidate the role of the ITO and the alumina underlayer on charge separation and transport (Figure 4). The photoinduced absorption (PIA) spectra of the Al_2_O_3_|ITO : ^NCN^CN_*x*_ electrode at 1.2 V vs RHE show a small feature assigned to holes in the ITO nanoparticles without 4‐MBA and a band at ≈650 nm assigned to trapped electrons in the ^NCN^CN_*x*_ upon addition of 4‐MBA (Figure 4a). These assignments are supported by spectroelectrochemical (SEC) measurements of Al_2_O_3_|ITO : ^NCN^CN_*x*_ and control experiments under different applied potentials (Figures S16 and S17). These data are consistent with previous reports as well as our photocurrent data (Figure 2a), showing that 4‐MBA is able to extract holes from the carbon nitride to leave electrons in the ^NCN^CN_*x*_.[^19^, ^44^] This observation suggests that the photoanode performance is likely limited by the extraction of electrons from the carbon nitride to the back contact rather than by hole transfer from the carbon nitride to 4‐MBA.[^51^, ^52^]

To study the electron extraction kinetics in the different samples, we examined the transient absorption (TAS, Figure 4b) and transient photocurrent response to a laser pulse (TPC, Figure 4c). TAS measurements of electron lifetimes, probed at 650 nm, confirm the faster charge extraction kinetics in Al_2_O_3_|ITO : ^NCN^CN_*x*_ compared to the long‐lived, deeply trapped photoelectrons in ^NCN^CN_*x*_ electrodes (Figure 4b).^[44]^ Electron extraction may begin on timescales faster than 10 μs after photoexcitation and continue for up to 1 ms (Figure 4e). As no increase in the electron population is observed in ^NCN^CN_*x*_ on this timescale, we suggest that 4‐MBA oxidation occurs on timescales faster than 10 μs. PIA measurements, carried out at open circuit voltage and at 1.2 V RHE, agree that photoaccumulated electrons are extracted more efficiently in Al_2_O_3_|ITO : ^NCN^CN_*x*_ than ^NCN^CN_*x*_ (Figure S18 and associated discussion). TPC kinetic measurements (Figure 4c) show the timescale and density of electron extraction from the electrode to the back contact. In the presence of ITO, the density of electrons extracted from the photoanode is substantially increased compared to bare ^NCN^CN_*x*_ electrodes, where a small amount of charge is extracted on the timescale of 10 μs–1 ms (Figure 4c inset). In Al_2_O_3_|ITO : ^NCN^CN_*x*_ and ITO : ^NCN^CN_*x*_ an additional 1 ms–1 s electron extraction process is observed. We assign the 10 μs–1 ms process in ^NCN^CN_*x*_ films to the extraction of electrons that are photogenerated adjacent to the carbon nitride/FTO back contact interface. The 1 ms–1 s electron extraction in Al_2_O_3_|ITO : ^NCN^CN_*x*_ is assigned to electrons that have been extracted from ^NCN^CN_*x*_ and transported through the ITO nanoparticle network (Figure 4e), supported by photoelectrochemical impedance spectroscopy (PEIS, Figure 4d), which is discussed further in the proceeding text. The timescale for electron transport from ^NCN^CN_*x*_ to the back contact is similar in both ITO‐containing photoanodes, with electron extraction half‐times in the order of 100 ms, consistent with PIA electron decay (Figure S18). The slow extraction half‐time observed in these ^NCN^CN_*x*_ electrodes suggests that electron transport is limited by the connectivity between ITO nanoparticles, with electrons likely forced to also travel through the low‐conductivity ^NCN^CN_*x*_ to reach the back contact.

This limiting role of ITO nanoparticles is also apparent from PEIS upon comparing the Nyquist plot of the ITO : ^NCN^CN_*x*_ film with that of the ^NCN^CN_*x*_ and ITO controls, measured at 0 V vs RHE, just before the photocurrent plateau (Figure 4d). Only one resistor‐capacitor (RC) process is observed in the ^NCN^CN_*x*_ electrode, which is assigned to electron transfer to the FTO (Figure S19), suggesting a hole transfer faster than the measurement timescale (>10 μs), in agreement with TAS data (Figure 4b, e). However, a new high‐frequency RC process is observed in the ITO‐containing electrodes (Figure 4d inset). Such a high‐frequency process is assigned to a highly resistive electron transfer from the ITO particles to the FTO taking place in the μs‐ms timescale. This is in agreement with the TPC data (Figure 4c, Figure S19), further confirming that hole transfer is faster than 10 μs. This further confirms that, although ITO nanoparticles are key in achieving high photocurrents, the PEC process is still limited by the charge transfer from ITO to the FTO (see extended discussion in the Supporting Information). Interestingly, the presence of the Al_2_O_3_ layer appears to significantly reduce the resistance of this high‐frequency electron transfer process, therefore improving the PEC performance of the Al_2_O_3_|ITO : ^NCN^CN_x_ photoelectrode. This improved contact has also been observed in dye‐sensitized solar cells and metal oxide photoelectrodes.[^53^, ^54^, ^55^]

The improved charge mobility observed upon adding alumina can be explained by this underlayer acting as a “tunnel barrier”, which reduces recombination losses at the FTO/ITO:^NCN^CN_x_ interface, thus enhancing effective carrier separation.^[53]^ This ultra‐thin insulating coating of Al_2_O_3_ allows for the photogenerated electrons in the semiconductor to tunnel to the conductive support. The Al_2_O_3_ layer thereby reduces electron‐hole recombination and the holes can subsequently only be quenched by reacting with the electrolyte solution.[^56^, ^57^] Crucially, the Al_2_O_3_ layer thickness must be carefully controlled to avoid photocurrent losses related to the insulating nature of the hole‐blocking material. Thus, the thinner the ALD‐deposited layer, the higher the photocurrent increase and the gradual photocurrent drop with increasingly thick alumina (Figure S3b).

Compared with previously reported CN_*x*_‐based electrodes, the as‐synthesized photoanodes exhibited benchmark photocurrents. To the best of our knowledge, the highest reported current of previous CN_*x*_ photoanodes reaches 660 μA cm^−2^ with a large excess of TEOA as the hole scavenger at 1.23 V vs RHE under AM 1.5G.^[58]^ The optimized Al_2_O_3_|ITO : ^NCN^CN_*x*_ electrode equals that recorded for glycerol oxidation and doubles it when selectively oxidizing 4‐MBA or ethanol under the same applied potential and light intensity, without the addition of any co‐catalyst on the surface and good conversion yields. ITO nanoparticles do not only increase the charge‐separation efficiency and the mechanical stability of the photoanode, but are also innocent towards the studied organic oxidation (Figure S4b, Table S1), as opposed to TiO_2_, another generally used metal oxide that is employed as an electron‐collecting scaffold in CN_*x*_‐based electrodes.[^59^, ^60^] In addition, the estimated onset potential of the ^NCN^CN_*x*_ photoanodes of approximately −0.4 V vs RHE is among the lowest for CN_*x*_ electrodes in the presence of a hole scavenger.^[37]^ The average IPCE values of carbon nitride photoanodes span between 15 and 20 %,^[17]^ whereas our Al_2_O_3_|ITO : ^NCN^CN_*x*_ photoanode reached 60 %. Furthermore, the use of organic model molecules as oxidation substrates, and not simple sacrificial agents, shows the potential of this material for PEC organic synthesis and reforming of waste materials, a field that has recently attracted attention.[^61^, ^62^]

## Conclusion

We have introduced a method to assemble versatile and high‐performance CN_*x*_ photoanodes for organic alcohol oxidation. The concurrent usage of ITO nanoparticles as conductive binding agent and a 1.8 Å thick alumina layer between the conductive glass support and the CN_x_ photocatalyst reached a high photocurrent for photoanodes using ^NCN^CN_*x*_ as the sole photoabsorber. A low onset potential was also achieved, which is comparable to state‐of‐the‐art metal‐oxide‐based photoanodes. Overall, our PIA, TAS, TPC, and PEIS data show that the ITO nanoparticles increase the efficiency of electron transport from ^NCN^CN_*x*_ to the back contact, whilst the alumina layer reduces the electrical resistance between the ITO nanoparticles and the back contact, reducing recombination and enhancing catalytic performance. 4‐MBA oxidation appears to occur on timescales faster than 10 μs, with electron transfer from ^NCN^CN_*x*_ to ITO occurring on timescales between 10 μs and 1 ms. Transport to the back contact then occurs in 1 ms to 1 s. Future analysis will further clarify the beneficial effect of the alumina layer and ITO. Additional studies are underway to improve the durability of the electrode over long illumination time, which may allow them to compete with other well‐established metal oxides, and to broaden the reaction scope by addition of a co‐catalyst.

## Conflict of interest

The authors declare no conflict of interest.

1

## Supporting information

As a service to our authors and readers, this journal provides supporting information supplied by the authors. Such materials are peer reviewed and may be re‐organized for online delivery, but are not copy‐edited or typeset. Technical support issues arising from supporting information (other than missing files) should be addressed to the authors.

Supporting Information

## Data Availability

The data that support the findings of this study are openly available at the Cambridge Data repository at repository: https://doi.org/10.17863/CAM.89481, reference number 89481.
