# Supplementary material for: Rational Design of Carbon Nitride Photoelectrodes with High Activity Toward Organic Oxidations
Source: Angew Chem Int Ed Engl. 2022 Nov 15;61(50):e202211587. doi: 10.1002/anie.202211587 (PMC10099510; doi:10.1002/anie.202211587)
Supplement: Supplementary file 1 — Supporting Information [file ANIE-61-0-s001.pdf]

Angewandte Chemie
Eine Zeitschrift der Gesellschaft Deutscher Chemiker

Supporting Information

Rational Design of Carbon Nitride Photoelectrodes with High Activity Toward Organic Oxidations

C. Pulignani, C. A. Mesa, S. A. J. Hillman, T. Uekert, S. Giménez, J. R. Durrant*, E. Reisner**

Supporting Information
©Wiley-VCH 2021
69451 Weinheim, Germany

Rational Design of Carbon Nitride Photoelectrodes with High Activity Toward Organic Oxidations

Carolina Pulignani,[a] Camilo A. Mesa,[b] Sam A. J. Hillman,[c] Taylor Uekert,[a] Sixto Giménez,*[b] James R. Durrant,*[c] and Erwin Reisner*[a]

DOI: 10.1002/anie.2021XXXXX

Table of Contents

Experimental Procedures S3

Material Synthesis S3

Photoelectrochemical Setup S3

Characterization S4

Product Analysis S4

Supplementary Figures S6

Supporting References S15

Authors contribution S15

Experimental Procedures

Material synthesis

4-Methyl benzyl alcohol (≥98% purity), ethanol (≥96% purity), methanol (HPLC grade), glycerol (HPLC grade), ethylene glycol, fluorine-doped tin oxide (FTO) glass, indium tin oxide (ITO) nanoparticles, melamine were purchased from Sigma-Aldrich.

Carbon nitride (CN_x) synthesis

Unfunctionalized CN_x was synthesized following a previously reported procedure.[1] Melamine (5 g) was heated at 550 °C for three hours (ramp rate 1 °C min^{-1}) under air. The obtained yellow powder (50% yield) was ground using a pestle and mortar.

$^{NCN}CN_x$ synthesis

Cyanamide-functionalized CN_x was synthesized by mixing the CN_x powder with potassium thiocyanate (weight ratio 1:2) and by heating the mixture firstly at 400 °C for an hour and then at 500 °C for 30 min (ramp rate 30°C min^{-1}) under Ar following a published procedure.[2] The powder was allowed to cool to room temperature, washed twice with H_2O and dried overnight at room temperature.

$^{NCN}CN_x$ Film Deposition

$^{NCN}CN_x$ was co-deposited with commercial ITO nanoparticles (~50 nm) on FTO-coated glass, adapting a literature procedure.[3] Different masks were prepared and used as a template for the deposition of the photo(electro)catalyst mix, either by cutting Parafilm with a drilling bill to obtain a 0.25 cm^2 , 0.33 cm^2, and 0.5 cm^2 circle. The Parafilm template was pressed onto the FTO side (1×2 cm) and slightly heated (45 s in a 120°C drying oven) to ensure uniform electrode size. $^{NCN}CN_x$ and ITO nanoparticles were combined at different weight ratios (50 mg total), dispersed in ethanol (1.1 mL) and ultrasonicated for 30 min. 5 μL of the mixture were drop-casted onto the templated FTO glass and allowed to dry in air producing 1 layer of catalyst. In this work, up to 2 layers of catalyst were studied. Once the last layer had dried, the mask was removed, and the electrodes were annealed at 250°C for 1 h under Ar (ramp rate 10°C min^{-1}).

ALD deposition of Al_2O_3 on FTO glass

A thin Al_2O_3 layer was grown by a commercial (AT-410, Anric Technologies) atomic layer deposition (ALD) on the FTO coated glass as a hole blocking layer. The precursors used were trimethylaluminum ($Al(CH_3)_3$) and water (H_2O) that were maintained at room temperature. A constant flow of 29 sccm of N_2 was used to carry the precursors to the reaction chamber preheated at 150 ºC. Each ALD cycle consisted of 3 pulses of $Al(CH_3)_3$, 11 s of N_2 purge, 2 pulses of H_2O and 13 s of N_2 purge. Each cycle was calibrated to produce an Al_2O_3 layer of 0.91 Å thick. Three different thickness were tested by depositing 2 layers (1.8 Å), 3 layers (2.7 Å), and 4 layers (3.6 Å) of Al_2O_3. The highest performance was achieved with the thinner layer (see Figure S3b), which was employed for all sample in this work, unless stated otherwise.

Photoelectrochemical Set-Up

A single compartment cell in a three-electrode configuration was filled with Na_2SO_4 electrolyte (0.1 M, 9 mL, pH 7) and the substrate of interest (i.e., 4-methylbenzyl alcohol (4-MBA), ethylene glycol (EG)) in a 50 mM concentration. For methanol, ethanol, and glycerol a 10% v/v solution was tested. Ag/AgCl in saturated KCl was used as reference electrode and Pt (Pt mesh supported on a Pt wire) as the counter electrode. After purging the cell with N_2 (~15 min), linear sweep voltammograms were performed (LSVs), applying a potential from -0.2 to +1.6 V vs. RHE at a rate of 10 mV s^{-1} under chopped simulated solar light (AM 1.5G, 100 mW cm^{-2}, 10 s on/off intervals). All measurements were conducted on three separate batches of samples, to verify reproducibility and to calculate standard deviation.
Photoelectrochemical impedance spectroscopy (PEIS) measurements were performed on a PGSTAT302N potentiostat (Metrohm-Autolab, The Netherlands) using the same conditions as the photoelectrochemical tests, but under a constant flow of Ar. PEIS measurements were carried out at selected applied potentials with a sinusoidal perturbation of 10 mV and a frequency range from 100 kHz to 0.1 Hz.

Characterization

UV-Vis spectroscopy. UV-vis spectra were recorded on a Varian Cary 60 UV-Vis spectrophotometer using a diffuse reflectance accessory (for powder and panel samples). The measured diffuse reflectance of the mixture is then inverted directly by the program using the Kubelka–Munk theory.

FT-IR spectroscopy. FT-IR spectra were recorded on a ThermoScientific Nicolet iS50 spectrometer. The Omnic software was used for analysis.

Electron microscopy. Scanning electron microscopy (SEM) was conducted on a TESCAN MIRA3 FEG-SEM. Samples were sputter-coated with a 10 nm layer of Cr (for SEM coupled with energy-dispersive X-ray spectroscopy, or EDX) prior to microscopy. Transmission electron microscopy (TEM) was conducted on a Thermo Schientific (FEI) Talos F200X G2 TEM. All samples were prepared by scratching the drop casted catalyst mixture from the FTO glass post-annealing. The mixture was then dispersed in ethanol (low concentration) and drop-cast onto carbon-coated Cu TEM grids and allowed to dry before use.

X-ray photoelectron spectroscopy (XPS). XPS analysis was carried out using a Escalab 250XI spectrometer from Thermo Fisher Scientific (West Sussex, UK). The instrument was operating in constant analyzer energy mode. A monochromatic Al-K_{α} source (1486.74 eV), *a* flood gun for charge neutralization and 300um of spot size were used. Survey scans were acquired using pass energy of 100 eV, 3 scans were recorded using 0.5 eV step size and dwell of 50ms. Two spectra at room temperature from different areas of the sample were acquired and then results were averaged.

Incident photon-to-current efficiency (IPCE) measurements. IPCE of the selected electrodes was determined using a New-port Oriel 66881 setup, on Oriel 74000 Cornerstone monochromator to selector the wavelength, with a full width at half maximum (FWHM) of 15 nm. The wavelength was typically varied between 300-500nm in 25 nm steps. An Air Mass 1.5 Global (AM 1.5G) solar filter was employed. The light intensity at different wavelengths was determined using a Thorlabs PM100D thermal power meter with a Thorlabs S302C thermal power sensor. The resulting photocurrent was measured on an IviumStat potentiate, with the sample as the working electrode, an Ag/AgCl/KCl (sat) reference electrode and platinum as counter electrode. All the measurements reported herein were performed in a 50 mM 4-MBA 0.1 M Na_2SO_4, with an external applied bias of 0.65 V vs Ag/AgCl (1.25 V vs RHE, following Equation 1).

$$E_{RHE} = E_{Ag/AgCl} + 0.059 * pH + E^0_{Ag/AgCl} \qquad \textit{Equation 1}$$

The IPCE values were determined using Equation 2, where *h* is the Planck constant, c is the speed of light, *J* is the photocurrent density, *e* is the electron charge and P_λ is the wavelength-dependent light intensity flux.

$$IPCE = \frac{hcJ}{eP_\lambda} \qquad \textit{Equation 2}$$

Faradaic efficiency (FE%). The FE of the photoelectrodes was determined by comparing the moles of products (mol_i) to the total charge passed (*Q*), as shown in Equation 3. The equivalent charge used per molecule converted is noted z=2, while F is the Faraday constant.

$$FE\ (\%) = \frac{mol_i * z * F}{Q} * 100 \qquad \textit{Equation3}$$

The total charge was obtained by integration of the recorded current (*I*) over the duration (*t*) of the chronoamperometry, illustrated in Eq. 4, where t_0 and t_e a represent the beginning and end-points of the measurement.

$$Q = \int_{t_0}^{t_e} I(t)\, dt \qquad \textit{Equation 4}$$

All samples were measured in a 3-electrode setup with the sample as working electrode, Pt mesh counter electrode and an Ag/AgCl (3M KCl) reference electrode. Samples were immersed into approximately 10 mL of 0.1 M Na_2SO_4, with or without 50 mM 4-MBA as described in the text / figure captions. The quartz cell was sealed and degassed for 30 minutes with nitrogen gas prior to measurements.

Spectroelectrochemistry: All SEC experiments were carried out in the absence of 4-MBA. JV curves were measured using an Ivium Vertex potentiostat at a sweep speed of 10 mVs^{-1}. Absorbance changes were measured using an in-house setup. The probe beam was generated using a Tungsten lamp (Thorlabs SLS201L/M with color-temperature balancing filter FGT05165 and collimation package attached), focused onto the sample and then directed into a liquid light guide (Thorlabs LLG5-4Z, 5 mm diameter, 420-2000 nm). The light was collected using a CCD operating at -80 degrees Celsius (Oxford Instruments, iDUS 420A-BEX2-DD). Absorbance

changes and JV curves were measured simultaneously, with time synchronization and data collection managed using an in-house LabView program.

PIA, TAS and TPC: Unless stated otherwise, all measurements were carried out in the presence of 50 mM 4-MBA. Unless stated otherwise, a potential of 1.2 V vs RHE (0.6 V vs Ag/AgCl) was applied using an Metrohm Autolab PGSTAT 101 operating in chronoamperometry mode. PIA and TPC data were collected simultaneously using an in-house setup controlled using a LabView program. TPC data was collected using a Tektronics DPO-2012B digital phosphor oscilloscope connected directly to the potentiostat. PIA/TAS spectra and kinetics were measured in transmission mode using an in-house setup. The probe beam used for PIA measurements was produced using white light from a 100 W Quartz Tungsten Halogen lamp (Bentham IL1). The white light was passed through an adjustable monochromator, followed by collimating, and focusing lenses and an iris. After transmittance through the sample the beam was passed through further collimating and focusing lenses, a second monochromator and a final focusing lens before incidence on a silicon photodiode (Hamamatsu S3071). The probe beam wavelength was incremented in steps of 50 nm by adjusting both monochromators. We note that the amplitudes of the PIA kinetics are not comparable between Figures 13.a and 13.b as the magnitude of the absorbance change is very sensitive to the sample history.

LED excitation: Samples were excited with a 365 nm LED (using a TTi QL564P DC power supply) which was directed onto the sample using a liquid light guide (3.2 mW cm^{-2} incident on the sample). In all cases, photoelectrodes were illuminated from the front (i.e. from the solvent side, not through the FTO back contact) for two seconds. A software-controlled MOSFET connected to the power supply in series with the LED was used to switch on the LED in 2 second pulses whilst simultaneously triggering data acquisition. Optical data was collected using an NI-USB-6361 National Instrument DAQ card.

Laser excitation: Samples were excited from the front side with a single 355 nm laser pulse from a Nd:YAG laser (OPOTEK Opolette 355 II, 7 ns pulse width) at an incident intensity of 540 µJ cm^{-2}. The beam was transmitted through a liquid light guide before incidence on the sample. To get the 100 ns – 10 s time range shown in the main text, each sample was excited several times with different timebase settings on the scope. The overlapping TPC traces were then stitched together. The TPC was monitored between pulses to ensure that each sample was given sufficient time to return to its unexcited state between excitation pulses. Optical data was collected on the 5 µs -1 ms timescale using a Tektronics DPO-2012B digital phosphor oscilloscope after being passed through an optical transient amplifier (Costronics 2004). Optical data on the 1 ms – 0.1 s timescale was collected using a NI-USB-6361 National Instrument DAQ card.

Product analysis

HPLC analysis. All analyses were conducted on a Waters Breeze system equipped with refractive index (RID-2414) and diode array UV–vis (λ = 210 and 254 nm) detectors. The 4-MBA oxidation product was identified and quantified with a C18 column at 40 °C column temperature. Samples were analyzed in the isocratic flow mode (flow rate 0.5 mL min^{-1}, H_2O:MeCN). Glycerol oxidation products were analyzed with an Ion-Exclusion ROA-Organic Acid H+ (8%) column at 70 °C. Samples were analyzed in the isocratic flow mode (flow rate 0.5 mL min^{-1}, 0.005 M H_2SO_4 HPLC water). Calibrations were conducted with external standards for both substrates.

Ionic Chromatography (IC). IC was performed to identify and quantify formate and acetate formation for MeOH, EtOH and glycerol oxidation. The analysis was performed with a 882 Metrohm Compact IC Plus using 3.2 mM Na_2CO_3 and 1 mM $NaHCO_3$ as an eluent. Calibration was conducted with external standards.

NMR spectroscopy. ^{1}H NMR spectroscopy was used to identify oxidation products of ethylene glycol oxidation. NMR spectra were collected with a Bruker 400 MHz Neo Prodigy spectrometer, with deuterated water as solvent at room temperature.

Supplementary Figures

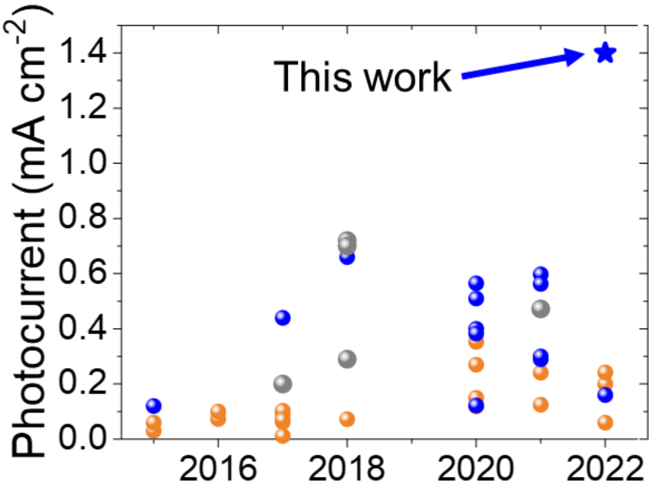

Figure S1. Most-relevant PEC performance of CN_x-based photoanodes selected from ref 4-16. The photocurrent response was measured at 1.23 V vs RHE applied potential with (blue dots) and without (orange dots) a hole scavenger. Photoanodes made by mixing CN_x with other metal oxide photoabsorber are depicted in grey dots.

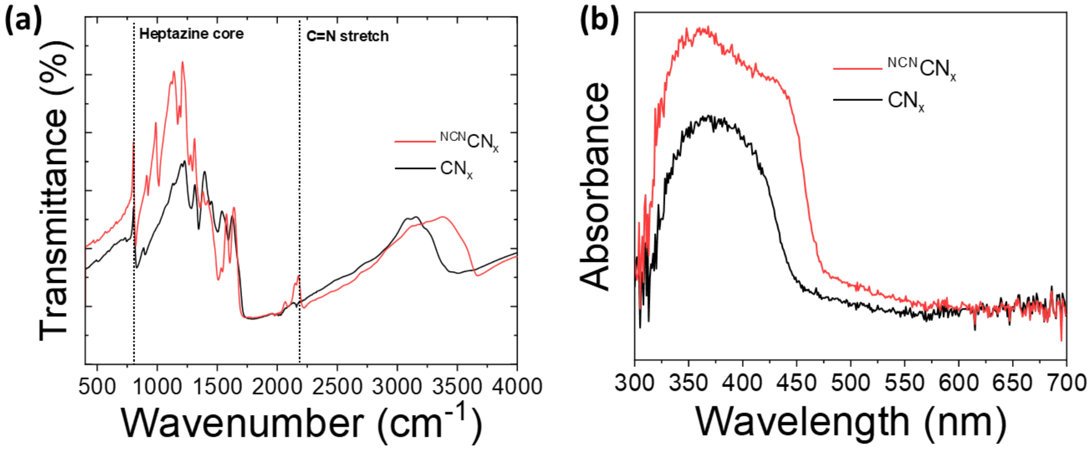

Figure S2. (a) IR and (b) UV-Vis analysis of CN_x (black trace) and $^{NCN}CN_x$ (red trace).

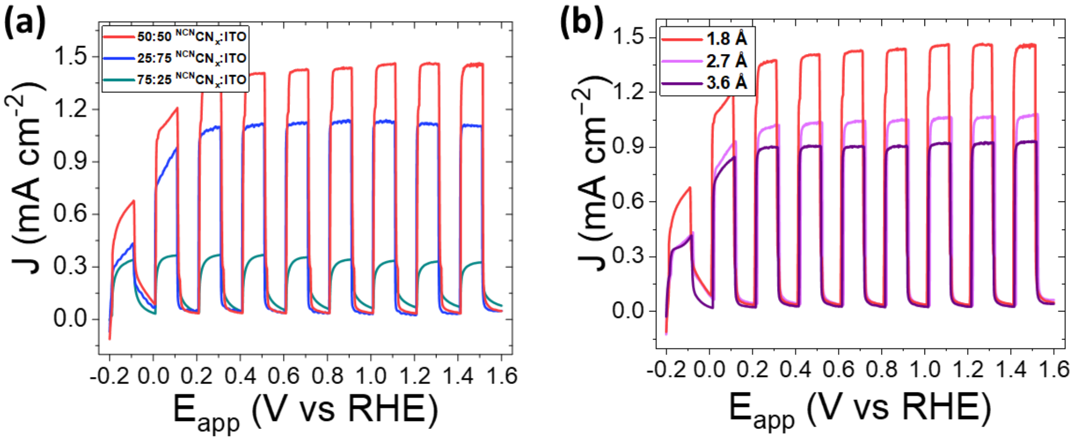

Figure S3. (a) Photocurrent optimization from oxidation of 4-MBA for co-deposited Al_2O_3|ITO:$^{NCN}CN_x$ photoelectrodes prepared with different weight ratios: 50:50 (red trace), 75:25 (green trace), and 25:75 (blue trace). (b) Photocurrent optimization for Al_2O_3|ITO:$^{NCN}CN_x$ photoelectrodes prepared 50:50 weight ratio and with different Al_2O_3 thickness. Conditions: 0.1 M aq. Na_2SO_4 (9 mL, pH 7), substrate (50 mM), scan rate (10 mV s^{-1}), chopped simulated solar light (AM 1.5G, 100 mW cm^{-2}), N_2 atmosphere, room temperature.

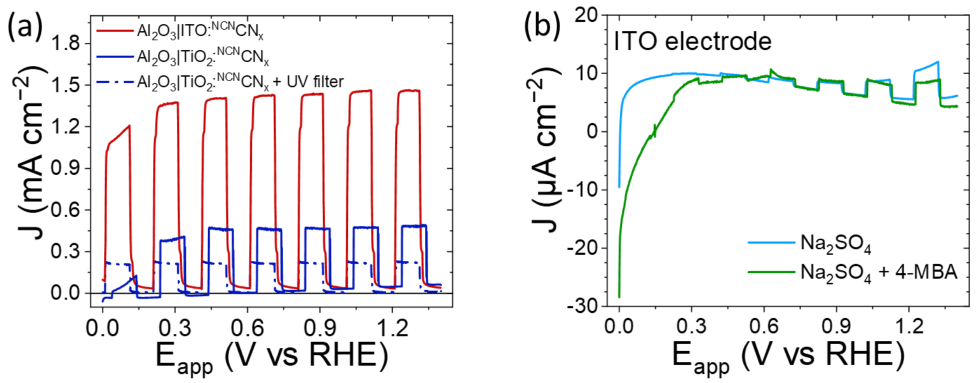

Figure S4. (a) LSVs of ITO electrodes (area 0.25 cm^{-2}) with (green trace) and without (light blue trace) the addition of 50 mM 4-MBA. (B) LSVs of Al_2O_3|ITO:$^{NCN}CN_x$ (red trace) and Al_2O_3|TiO_2:$^{NCN}CN_x$ (blue trace). Conditions: 0.1 M aq. Na_2SO_4 (9 mL, pH 7), 4-MBA (50 mM), scan rate (10 mV s^{-1}), chopped simulated solar light (AM 1.5G, 100 mW cm^{-2}), N_2 atmosphere.

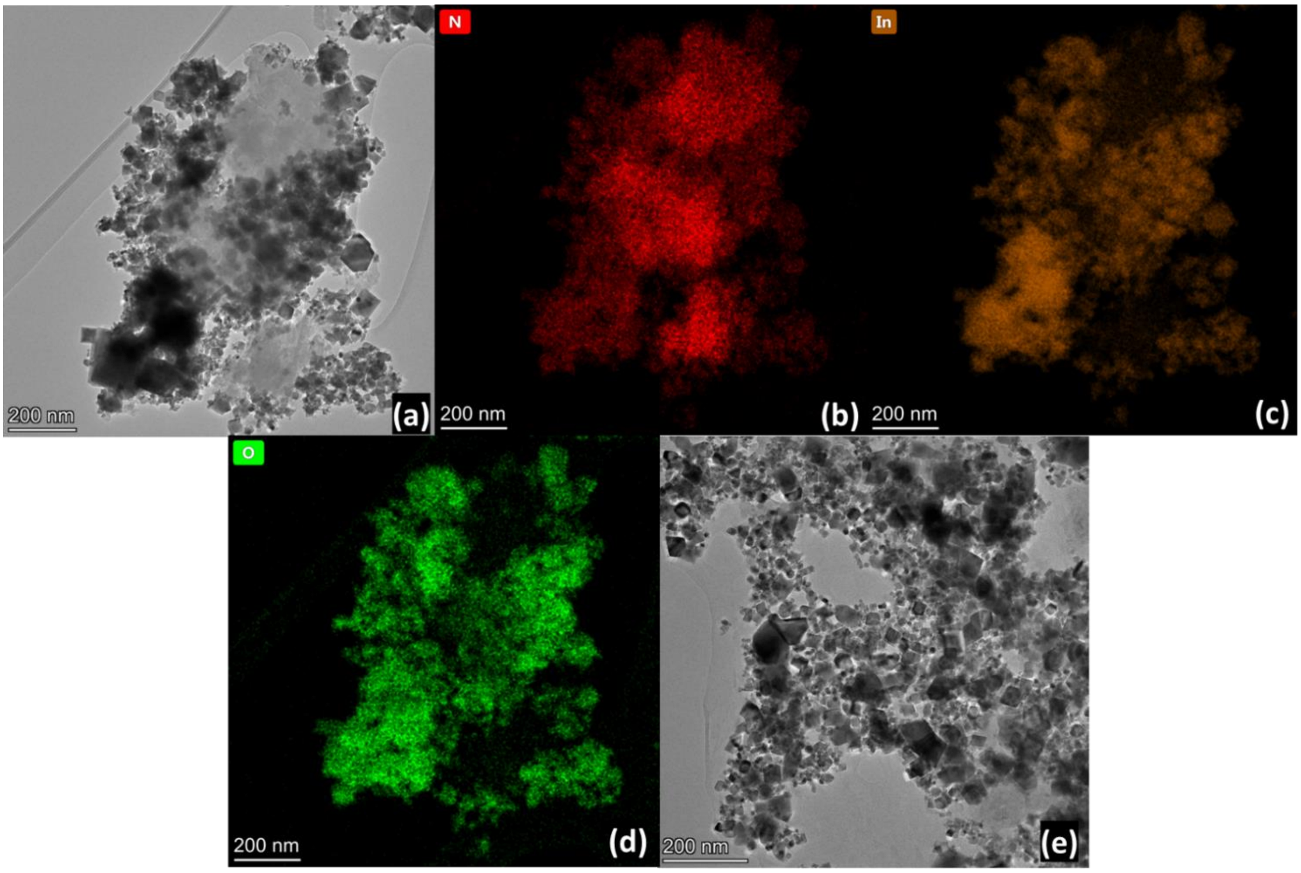

Figure S5. (a,e) TEM images of ITO:$^{NCN}CN_x$ from Al_2O_3|ITO:$^{NCN}CN^x$ photoelectrodes. TEM EDS mapping of ITO:$^{NCN}CN_x$ mixture from the relative electrodes showing the elemental distribution of (b) nitrogen, (c) indium, and (d) oxygen. images of $^{NCN}CN_x$ photoelectrodes: top views at a magnification of (a) 2000×, (b) 4000x, and (c) 10000×. Samples were sputter-coated with a 10 nm layer of Cr prior to measurement.

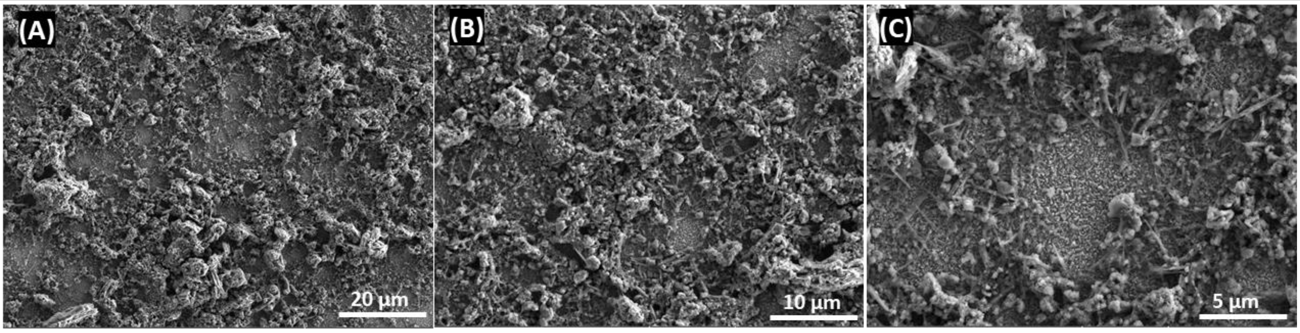

Figure S6. SEM images of $^{NCN}CN_x$ photoelectrodes: top views at a magnification of (a) 2000×, (b) 4000x, and (c) 10000×. Samples were sputter-coated with a 10 nm layer of Cr prior to measurement.

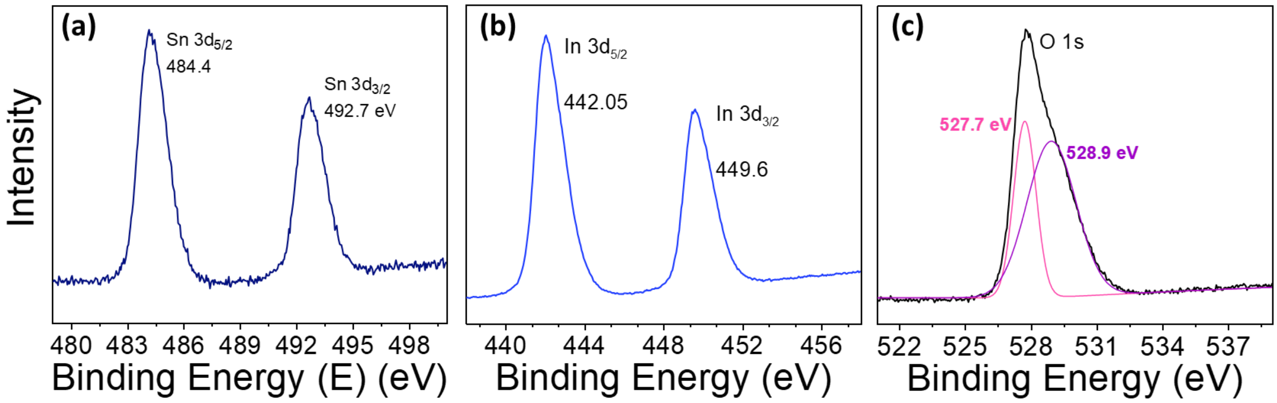

Figure S7. XPS spectra of (a) Sn_{3d}, (b) In_{3d}, and (c) O_{1s} edges for the $Al_2O_3|^{NCN}CN_x$-ITO electrodes.

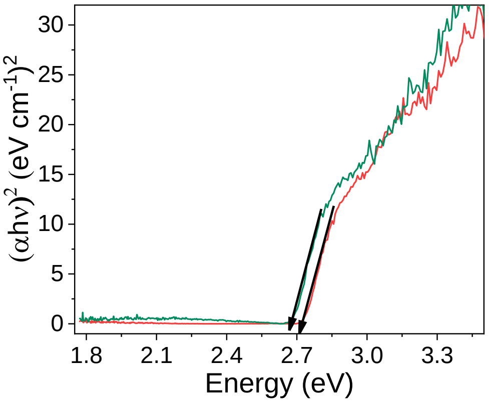

Figure S8. Tauc Plot of the $^{NCN}CN_x$ powder (green trace) and $Al_2O_3|ITO{:}^{NCN}CN_x$ electrodes (red trace).

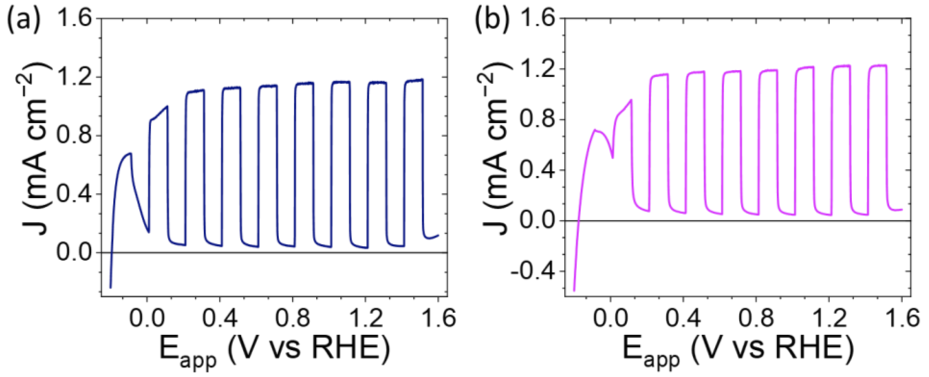

Figure S9. (a) LSV of Al_2O_3|ITO:$^{NCN}CN_x$ photoelectrodes illuminated from the back. (b) LSV of Al_2O_3|ITO:$^{NCN}CN_x$ photoelectrodes after 10 min of pre-illumination. Conditions: 0.1 M Na_2SO_4 pH 7 electrolyte solution, 50 mM 4-MBA, scan rate (10 mV s^{-1}), chopped simulated solar light (AM 1.5G, 100 mW cm^{-2}), N_2 atmosphere, room temperature.

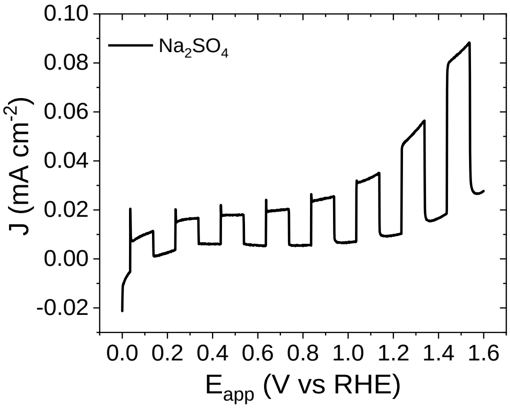

Figure S10. LSV of Al_2O_3|ITO:$^{NCN}CN_x$ photoelectrodes in 0.1 M Na_2SO_4 pH 7 electrolyte solution, scan rate (10 mV s^{-1}), chopped simulated solar light (AM 1.5G, 100 mW cm^{-2}), N_2 atmosphere.

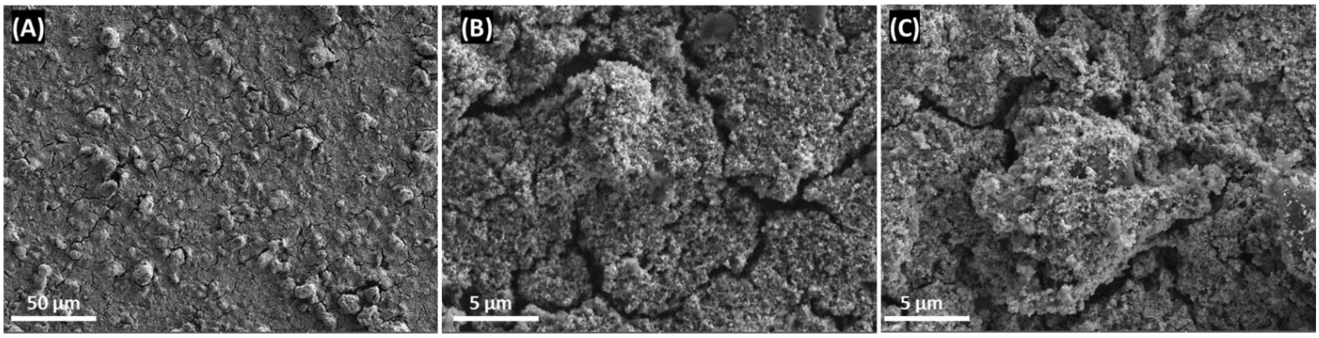

Figure S11. SEM images of Al_2O_3|ITO:$^{NCN}CN_x$ photoelectrodes after 16 h illumination, with an applied potential of 1.23 V vs RHE. Top views at a magnification of (a) 1000×, and (b,c) 10000×. Samples were sputter-coated with a 10 nm layer of Cr prior to measurement.

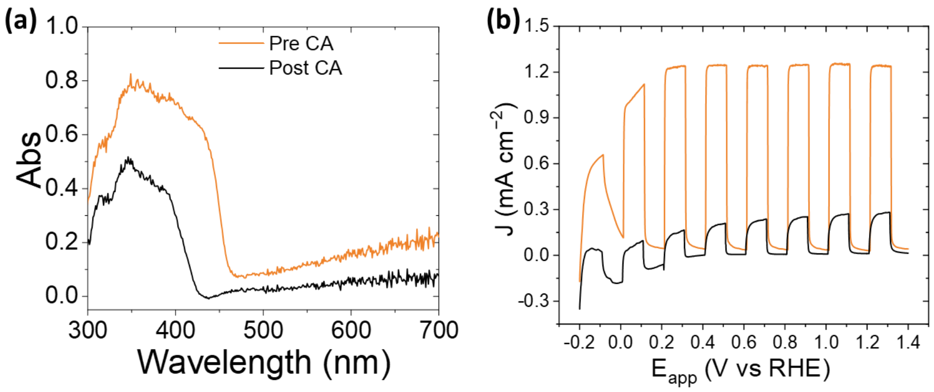

Figure S12. (a) UV-Vis spectra of Al_2O_3|ITO:$^{NCN}CN_x$ films before (orange trace) and after (black trace) 16h illumination, with an applied potential of 1.23 V vs RHE. (b) LSVs of Al_2O_3|ITO:$^{NCN}CN_x$ films before (orange trace) and after (black trace) after 16h under an applied potential of 1.25 V vs RHE in the dark. PEC conditions: 0.1 M aq. Na_2SO_4 (9 mL, pH 7), 4-MBA (50 mM), scan rate (10 mV s^{-1}), chopped simulated solar light (AM 1.5G, 100 mW cm^{-2}), N_2 atmosphere.

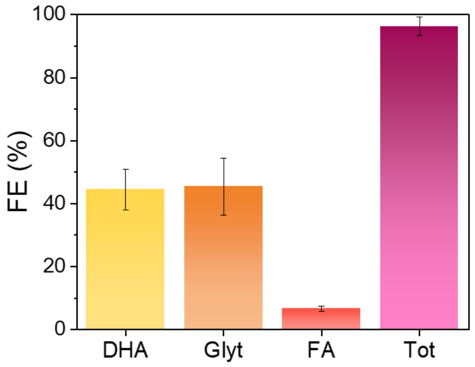

Figure S13. FE (%) of glycerol photoelectrochemical oxidation with Al_2O_3|ITO:$^{NCN}CN_x$ electrodes. Main product identified: dihydroxyacetone (DHA), glyceraldehyde (glyt), and formic acid (FA).

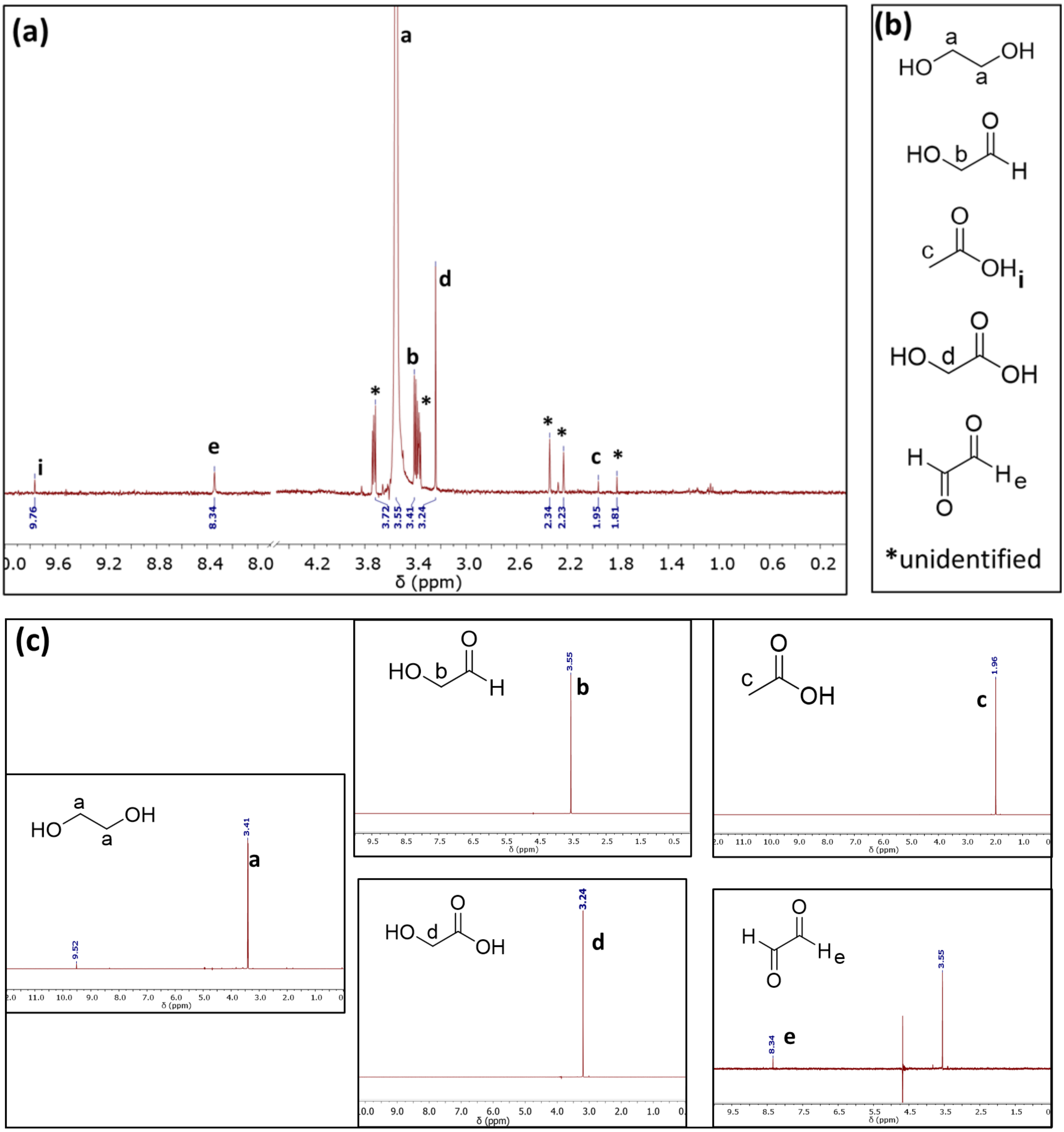

Figure S14. (a) ^{1}H-NMR spectra of EG solution after 21h PEC in D_2O. (b) Chemical structure of the starting material (top) and the identified oxidation products, namely glycolaldehyde, acetic acid, glycolic acid, and glyoxal. (c) ^{1}H-NMR spectra of the pure identified oxidation products in D_2O. PEC conditions: 21h under simulated solar light illumination (AM 1.5G, 100 mW cm^{-2}, 25°C, 50 min on/10 min off), 50 mM EG 0.1 M Na_2SO_4 solution (pH 7).

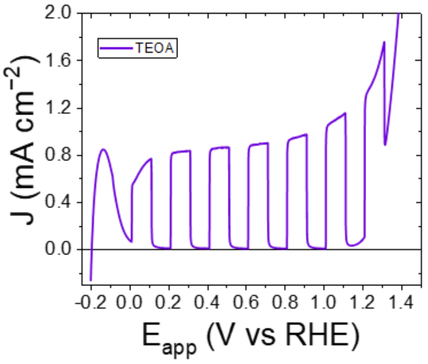

Figure S15. LSVs of Al_2O_3|ITO:$^{NCN}CN_x$ photoanode. Conditions: 10% v/v TEOA in 0.1 M Na_2SO_4 (pH 7), scan rate (10 mV s^{-1}), chopped simulated solar light (AM 1.5G, 100 mW cm^{-2}), N_2 atmosphere

.

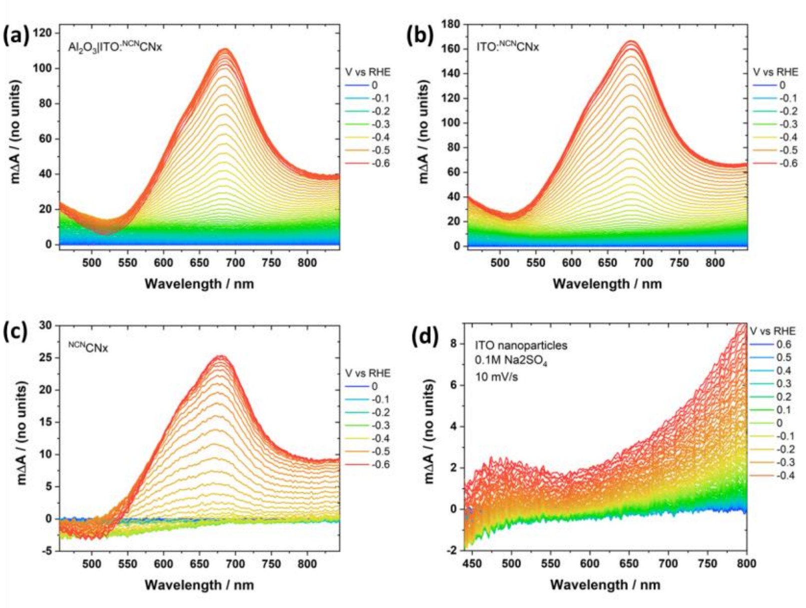

Figure S16. Absorbance difference spectra at increasingly negative applied bias for: (a) Al_2O_3|ITO:$^{NCN}CN_x$, (b) ITO:$^{NCN}CN_x$, (c) $^{NCN}CN_x$, and (d) ITO nanoparticle photoanodes. All samples were measured in a 0.1 M Na_2SO_4 solution in nitrogen with a scan rate of 10 mVs^{-1}.

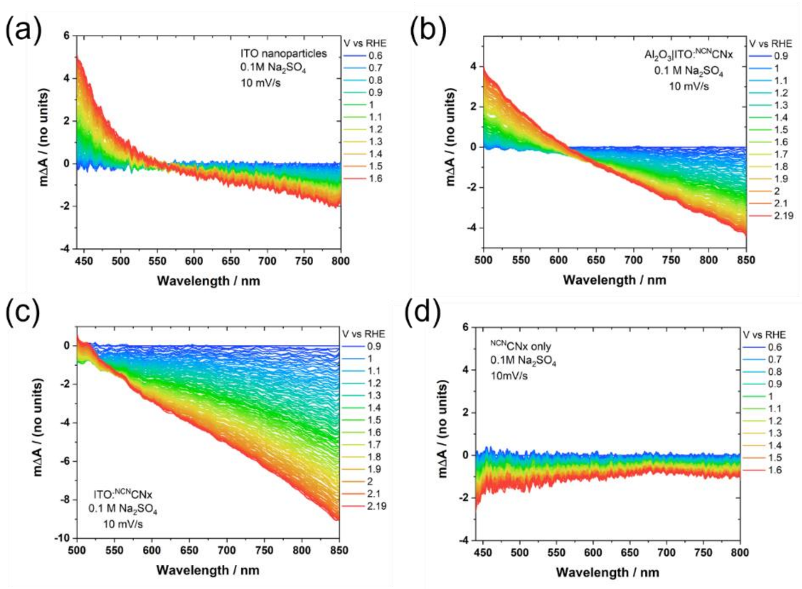

Figure S17. Absorbance difference spectra at increasingly positive applied bias for: (a) ITO nanoparticle, (b) Al_2O_3|ITO:$^{NCN}CN_x$, (c) $^{NCN}CN_x$; (d) $^{NCN}CN_x$ electrodes. All samples were measured in a 0.1 M Na_2SO_4 solution in nitrogen with a scan rate of 10 mVs^{-1}.

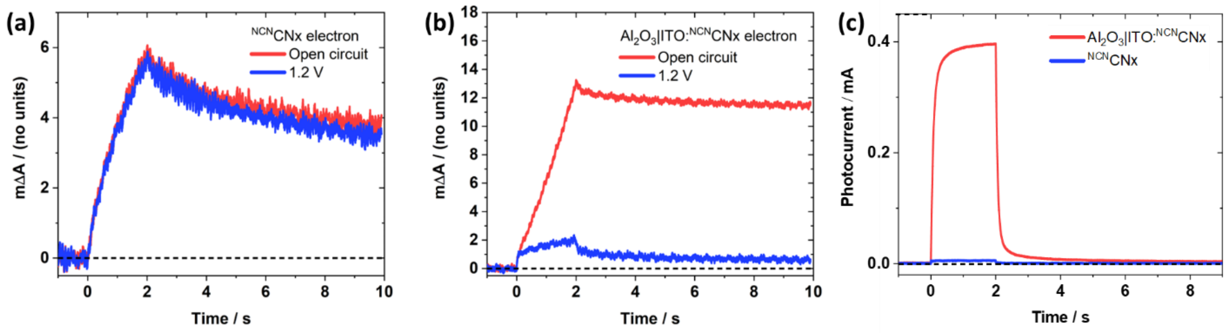

Figure S18. PIA decays of the electron population in the carbon nitride after 2 seconds of illumination with a 365 nm LED (3.2 mW cm^{-2}), probed at 650 nm. Data shown for an $^{NCN}CN_x$ electrode (a) and an Al_2O_3|ITO: $^{NCN}CN_x$ electrode (b) held at open circuit and at 1.2 V in solutions containing 0.1 M Na_2SO_4 and 50 mM 4-MBA. (c) Transient photocurrents for ^{NCN}CNx and Al_2O_3|ITO: ^{NCN}CNx samples held at 1.2V vs RHE, measured simultaneously with the optical data in (a) and (b).

Extended PIA/TAS/TPC discussion: Spectroelectrochemistry was first employed to identify the absorbance spectra of any charged species that might be present in the electrodes: $^{NCN}CN_x$, ITO: $^{NCN}CN_x$ and Al_2O_3|ITO:$^{NCN}CN_x$. Figure S17.b shows the change in absorbance of the Al_2O_3|ITO:$^{NCN}CN_x$ electrode under increasingly negative potentials relative to the sample's absorbance at 0 V RHE. Between 0 V and -0.3 V, a broad absorbance increase is observed which we assign to the presence of electrons in the ITO (Figure S16.b-d). At potentials beyond -0.3 V, a positive absorbance peak at 680 nm grows in concurrently with a substantial increase in current, which we assign to the presence of electrons in the carbon nitride. To study the electron extraction efficiency in the different samples, we examined the PIA kinetics of the carbon nitride electrons when $^{NCN}CN_x$ and Al_2O_3|ITO:$^{NCN}CN_x$ samples are held at open circuit and at 1.2 V RHE (Figure S17). In all four kinetic traces, a substantial proportion of the electron population decays slowly (half-lives > 10 seconds) after the LED is switched off. This is consistent with other reports of long-lived, trapped photoelectrons in NCN-terminated carbon nitrides. Whilst these electrons have previously been shown to reside in shallow, catalytically active trap sites.[17,18] Transient photocurrent data measured simultaneously with the optical data (Figure S17c) shows that the slowly-decaying electron population observed in these photoanodes does not contribute to the photocurrent: whilst these electrons may exist primarily in shallow trap states, they are still too deeply trapped to be efficiently electrically extracted. The bias independence of the PIA electron kinetics in Figure S18.a confirms that electron extraction is inefficient in the bare ^{NCN}CNx sample. In contrast, the Al_2O_3|ITO:$^{NCN}CN_x$ PIA signal show a strong dependence on the applied potential, being strongly quenched by an applied bias of 1.2 V, which is indicative of efficient electron extraction mediated by the ITO nanoparticles (Figure S18b).

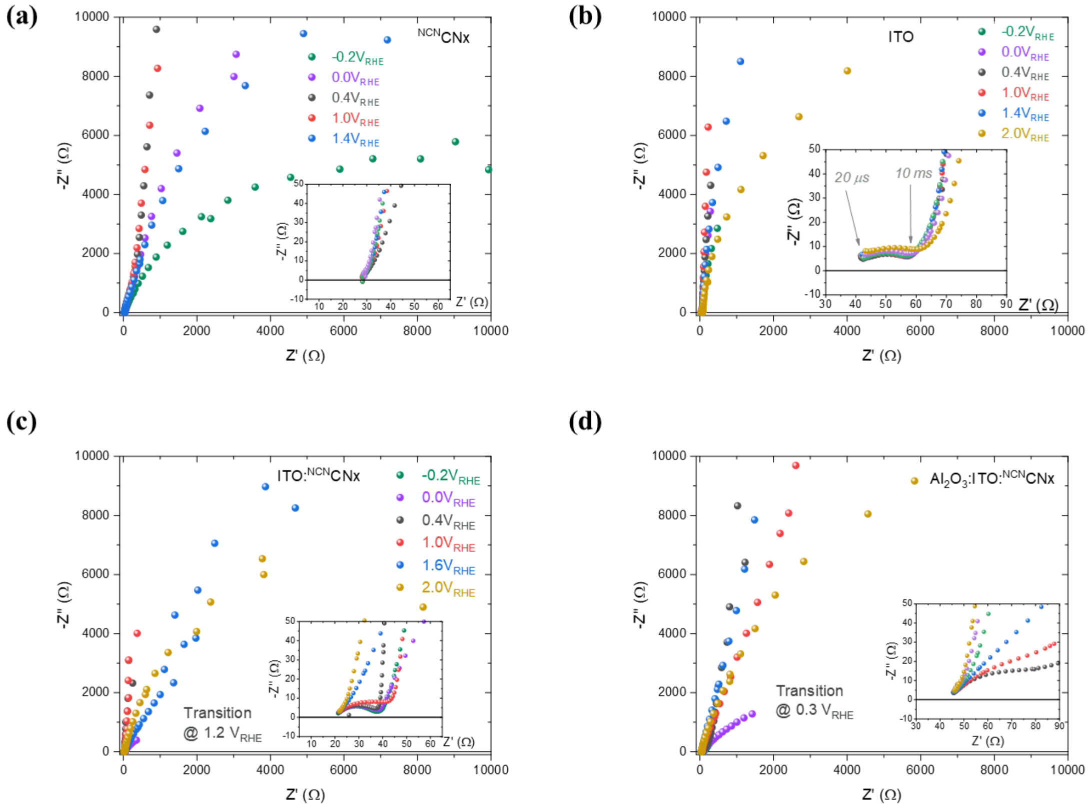

Figure S19. Nyquist plots of the (a) $^{NCN}CN_x$, (b) ITO, (c) $^{NCN}CN_x$:ITO blanks and the (d) Al_2O_3|ITO:$^{NCN}CN_x$ photoelectrodes measured as a function of the applied potential from -0.2V vs RHE to 2.0 V vs RHE under Ar atmosphere in 0.1 M aq. Na_2SO_4 (9 mL, pH 7), 4-MBA (50 mM) and solar light (AM 1.5G, 100 mW cm^{-2}). Frequency range used from 100 kHz to 0.1 Hz.

Extended PEIS discussion: Figure S19a shows the Nyquist plot for the $^{NCN}CN_X$ blank film as a function of the applied potential. It is apparent from this figure that there is only one semicircle, associated to one RC process. This RC process can be associated with a electron extraction process from $^{NCN}CN_X$ to the FTO back contact, given the PEC behavior shown in Figure 1.a in the manuscript. On the contrary, the ITO (Figure S19b) exhibits one high frequency semicircle and one low frequency semicircle, where the low-frequency semicircle shows a very high resistance that only starts decreasing at high applied potentials once dark current (charge injection) takes place (see dark onset potential in Figure S9). Interestingly, the high-frequency semicircle in ITO shown Figure S18b is independent of the applied potential suggesting that this process is not a charge transfer to the 4-MBA, but rather from ITO to FTO as observed previously in CoFe-prussian blue electrocatalysts on FTO.[19] Figure S19c shows, from -0.2 to 1.2 V vs RHE, 2 RC processes for the composite ITO: $^{NCN}CN_x$ photoelectrode similarly to the ITO alone. It is apparent from this figure, that in the potential range from -0.2 to 1.2 V vs RHE, both high and low-frequency RC processes behave similarly in both ITO and ITO: $^{NCN}CN_x$ electrodes the electron transfer from ITO to FTO dominates their JV curves. Thus, it can be concluded that the photoelectrochemical (PEC) behavior of the ITO: $^{NCN}CN_x$ photoelectrode is determined by the efficiency of charge injection from the ITO to the FTO. Above 1.2 V vs RHE, coinciding with the photocurrent plateau behavior shown in Figure 2a, orange data, in the manuscript, only one semicircle, associated to charge transfer to the 4-MBA, takes place, suggesting that the PEC behavior is no longer limited by the ITO/FTO interface, but rather by the electron extraction from the CN_x to the FTO. A similar behavior is observed for the Al_2O_3|$^{NCN}CN_x$:ITO photoelectrode, shown in Figure S19d, but with the transition from 2 to 1 RC processes limiting the PEC behavior at ~0.3 V vs RHE, suggesting that the role of the Al_2O_3 thin layer is to improve the electrical contact at the ITO/FTO interface.

Table S1. Control studies of $Al_2O_3|^{NCN}CN_x$:ITO electrodes. Conditions unless stated otherwise below: Chronoamperometry of at least 16 h under simulated solar light illumination (AM 1.5G, 100 mW cm^{-2}, 25°C, 50 min on/10 min off), 50 mM 4-MBA 0.1 M Na_2SO_4 solution (pH 7).

Photoanode	Conductive substrate	Light	Applied Potential (vs RHE)	Conversion (%)
**/**	/	1 Sun	/	0.11 ± 0.02
**ITO:$^{NCN}CN_x$**	Al_2O_3|FTO	/	1.23 V	0.38 ± 0.1
**ITO:$^{NCN}CN_x$**	Al_2O_3|FTO	1 Sun	0.6 V	17.7 ± 2.4
**ITO:$^{NCN}CN_x$**	Al_2O_3|FTO	1 Sun	/	4.6 ± 0.5

Supporting References

[1] J. Liu, Y. Liu, N. Liu, Y. Han, X. Zhang, H. Huang, Y. Lifshitz, S. T. Lee, J. Zhong, Z. Kang, *Science* **2015**, *347*, 970–974.

[2] V. W. H. Lau, I. Moudrakovski, T. Botari, S. Weinberger, M. B. Mesch, V. Duppel, J. Senker, V. Blum, B. V. Lotsch, *Nat. Commun.* **2016**, *7*, DOI 10.1038/ncomms12165.

[3] M. A. Bajada, S. Roy, J. Warnan, K. Abdiaziz, A. Wagner, M. M. Roessler, E. Reisner, *Angew. Chemie Int. Ed.* **2020**, *59*, 15633–15641.

[4] M. Volokh, G. Peng, J. Barrio, M. Shalom, *Angew. Chemie Int. Ed.* **2019**, *58*, 6138–6151.

[5] W. Xiong, F. Huang, R. Q. Zhang, *Sustain. Energy Fuels* **2020**, *4*, 485–503.

[6] M. Huang, H. Wang, W. Li, Y. L. Zhao, R. Q. Zhang, *J. Mater. Chem. A* **2020**, *8*, 24005–24012.

[7] J. Xia, N. Karjule, L. Abisdris, M. Volokh, M. Shalom, *Chem. Mater.* **2020**, *32*, 5845–5853.

[8] N. Karjule, J. Barrio, L. Xing, M. Volokh, M. Shalom, *Nano Lett.* **2020**, *20*, 4618–4624.

[9] J. Qin, J. Barrio, G. Peng, J. Tzadikov, L. Abisdris, M. Volokh, M. Shalom, *Nat. Commun.* **2020**, *11*, 1–9.

[10] N. Karjule, C. Singh, J. Barrio, J. Tzadikov, I. Liberman, M. Volokh, E. Palomares, I. Hod, M. Shalom, *Adv. Funct. Mater.* **2021**, *31*, 1–10.

[11] J. Xu, M. Shalom, *ACS Appl. Mater. Interfaces* **2016**, *8*, 13058–13063.

[12] A. Tashakory, N. Karjule, L. Abisdris, M. Volokh, M. Shalom, *Adv. Sustain. Syst.* **2021**, *2100005*, 1–7.

[13] N. Karjule, L. Abisdris, A. Azoulay, M. Volokh, M. Shalom, *Adv. Energy Sustain. Res.* **2022**, *2200035*, 2200035.

[14] X. Li, J. X. Jiawen Wang, B. Yuanxing Fang, Yidong Hou,[a] Xianzhi Fu,[a] Menny Shalom, and X. Wang, *ChemSusChem* **2022**, *15*.

[15] C. Adler, I. Krivtsov, D. Mitoraj, L. dos Santos-Gómez, S. García-Granda, C. Neumann, J. Kund, C. Kranz, B. Mizaikoff, A. Turchanin, R. Beranek, *ChemSusChem* **2021**, *14*, 2170–2179.

[16] X. Fan, Z. Wang, T. Lin, D. Du, M. Xiao, P. Chen, L. Wang, *Angew. Chemie Int. Ed.* **2022**, *e202204407*, DOI 10.1002/anie.202204407.

[17] H. Kasap, C. A. Caputo, B. C. M. Martindale, R. Godin, V. W. Lau, B. V Lotsch, J. R. Durrant, E. Reisner, *J. Am. Chem. Soc.* **2016**, *138*, 9138–9192.

[18] W. Yang, R. Godin, H. Kasap, B. Moss, Y. Dong, S. A. J. Hillman, L. Steier, E. Reisner, J. R. Durrant, *J. Am. Chem. Soc.* **2019**, *141*, 11219–11229.

[19] F. S. Hegner, I. Herraiz-Cardona, D. Cardenas-Morcoso, N. López, J. R. Galán-Mascarós, S. Gimenez, *ACS Appl. Mater. Interfaces* **2017**, *9*, 37671–37681.

Author Contributions

C.P. performed most of the experiments, material characterization, analyzed the data, and wrote the original draft of the manuscript. C.A.M. performed the ALD deposition, PEIS measurements and relative data analysis, took part in writing, and contributed to the design of the work. S.A.J.H. performed the spectroscopic measurements, relative data analysis, and contributed to the writing. T.U. contributed to the design of the work. J.D. supervised the spectroscopy studies. E.R. supervised the study and co-wrote the paper. All the authors discussed the results and reviewed the manuscript.
